# Resorbable screws versus pins for optimal transplant fixation (SPOT) in anterior cruciate ligament replacement with autologous hamstring grafts: rationale and design of a randomized, controlled, patient and investigator blinded trial [ISRCTN17384369]

**DOI:** 10.1186/1471-2482-5-1

**Published:** 2005-02-21

**Authors:** Dirk Stengel, Gerrit Matthes, Julia Seifert, Volker Tober, Sven Mutze, Grit Rademacher, Axel Ekkernkamp, Kai Bauwens, Michael Wich, Dirk Casper, the SPOT Group

**Affiliations:** 1 Department of Orthopaedic and Trauma Surgery, Unfallkrankenhaus Berlin Trauma Center, Warener Str 7, 12683 Berlin, Germany; 2 Department of Orthopaedic and Trauma Surgery, Sauerbruchstr., University Hospital of Greifswald, 17487 Greifswald, Germany; 3 Department of Clinical Epidemiology, Unfallkrankenhaus Berlin Trauma Center, Warener Str 7, 12683 Berlin, Germany; 4 Institute of Radiology, Unfallkrankenhaus Berlin Trauma Center, Warener Str 7, 12683 Berlin, Germany

## Abstract

**Background:**

Ruptures of the anterior cruciate ligament (ACL) are common injuries to the knee joint. Arthroscopic ACL replacement by autologous tendon grafts has established itself as a standard of care.

Data from both experimental and observational studies suggest that surgical reconstruction does not fully restore knee stability. Persisting anterior laxity may lead to recurrent episodes of giving-way and cartilage damage. This might at least in part depend on the method of graft fixation in the bony tunnels. Whereas resorbable screws are easy to handle, pins may better preserve graft tension. The objective of this study is to determine whether pinning of ACL grafts reduces residual anterior laxity six months after surgery as compared to screw fixation.

**Design/ Methods:**

SPOT is a randomised, controlled, patient and investigator blinded trial conducted at a single academic institution. Eligible patients are scheduled to arthroscopic ACL repair with triple-stranded hamstring grafts, conducted by a single, experienced surgeon. Intraoperatively, subjects willing to engage in this study will be randomised to transplant tethering with either resorbable screws or resorbable pins. No other changes apply to locally established treatment protocols. Patients and clinical investigators will remain blinded to the assigned fixation method until the six-month follow-up examination.

The primary outcome is the side-to-side (repaired to healthy knee) difference in anterior translation as measured by the KT-1000 arthrometer at a defined load (89 N) six months after surgery. A sample size of 54 patients will yield a power of 80% to detect a difference of 1.0 mm ± standard deviation 1.2 mm at a two-sided alpha of 5% with a t-test for independent samples.

Secondary outcomes (generic and disease-specific measures of quality of life, magnetic resonance imaging morphology of transplants and devices) will be handled in an exploratory fashion.

**Conclusion:**

SPOT aims at showing a reduction in anterior knee laxity after fixing ACL grafts by pins compared to screws.

## Background

Anterior cruciate ligament (ACL) rupture belongs to the most common musculoskeletal injuries in the western world. In the United States, 100,000 new cases occur each year, with 10% of all injuries leading to occupational disability [1]. In Germany, the prevalence of torn ACL among subjects between 20 and 35 years averages 0.4%. In the general population, the yearly incidence of ACL rupture reaches 32/100,000, but peaks to 70/100,000 in athletes [2].

An ACL deficient knee is at risk of developing secondary damage to the cartilage and is liable to undergo progressive intra-articular worsening. Roughly half of all acute ACL disruptions attend meniscus damage. Arthroscopic reconstruction surgery by autologous grafting emerged as the therapy of choice. Predictions call for 175,000 ACL replacements performed yearly in the United States. In Germany, around 50,000 patients undergo ACL repair each year.

Selecting the ideal graft remains an issue of debate. Randomized controlled trials suggest a lower degree of persistent laxity with bone-patellar-tendon-bone (BPTB) comparing with two-, three- or four-bundle hamstring (that is, semitendinosus and gracilis tendon) transplants (HT).

However, the biomechanical advantage does not frame higher patient satisfaction, or differences in scoring after long-term follow-up [3]. In contrast, harvesting BPTB grafts often produces notable donor site morbidity, and refractory kneeling pain [4,5].

**Figure 1:**
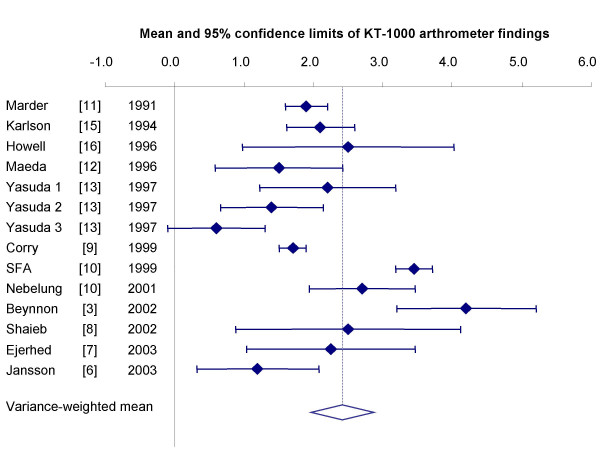
Persisting instability following ACL repair with HT autografts (KT-1000 measurements). Individual study results were weighted by their inverse variance to derive a common point estimate with 95% confidence interval (diamond).

Available data from randomized, quasi-randomized and uncontrolled trials signal a weighted mean difference of 2.28 mm (95% confidence interval [CI] 1.83 – 2.73 mm) in anterior laxity between the injured and healthy knee with HT reconstruction (see Figure 1) [3,6-16].

Many features contribute to an unsatisfactory or failed ACL replacement, for example, imprecise tunnel positioning, the presence of degenerative changes, or the onset of arthrofibrosis.

**Figure 2:**
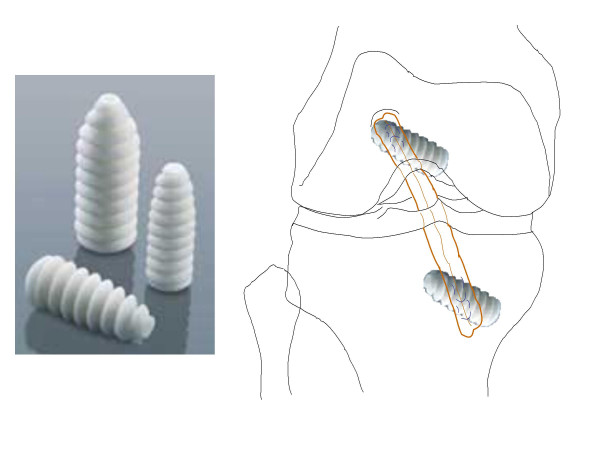
Appearance of the BioCryl^® ^screws (left, courtesy of DePuy Mitek), and their positioning (right).

The choice of tibial graft fixation affects later stability. The intact ACL has a tensile strength around 2200 N. To avoid loosening, the graft must be fixed under firm traction (around 40 N), with the knee in a smoothly flexed position. A common way to anchor the tibial end of the graft is by titanium or biodegradable interference screws, for example, the BioCryl^® ^(DePuy Mitek) screw that contains both resorbable poly-L-lactid and osteoconductive tricalcium phosphate (see Figure 2).

**Figure 3:**
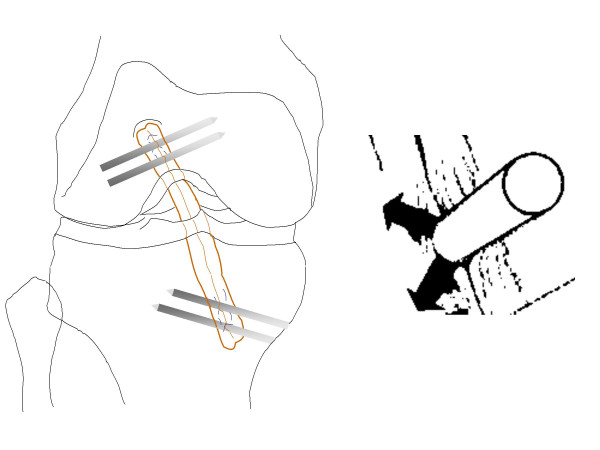
Left: Positioning of RigidFix^® ^pins. Right: splicing of graft bundles leading to close adherence to the surrounding bone.

However, in a recent biomechanical study, extracortical fixation devices like the EndoButton^® ^(Smith & Nephew) or RigidFix^® ^(DePuy Mitek) provided better strength than did the interference screws [17]. The possible advantage of RigidFix^® ^over other tethering methods is a splicing of strands, tightening the contact between the tendon surface and the bony tunnel over the entire graft circumference (see Figure 3).

## Methods/ Design

### Objectives

The present study aims at comparing later laxity in subjects undergoing arthroscopic anterior cruciate ligament replacement with either RigidFix^® ^pinning or BioCryl^® ^screwing of HT grafts. Both implants are CE approved, and were introduced to ACL-repair in Germany in 2002.

We have secondary objectives in imagining graft incorporation by MRI-scanning, functional results, residual pain, resumption of occupational and leisure activity, and quality of life by generic and disease-specific questionnaires.

### Primary endpoint

We pose the primary hypothesis that the RigidFix^® ^system preserves graft tension gained during surgery, and leads to lower KT-1000 arthrometer side-to-side differences than the BioCryl^® ^screw after six months of follow-up.

Specifically, we will test the hypothesis that RigidFix^® ^decreases the average difference gained with interference screws by 1.0 ± standard deviation 1.2 mm. The investigators consider this difference clinically sound, important, and measurable by KT-1000 arthrometer testing. Twenty-four subjects a treatment arm will allow for detecting this difference with an 80% chance at a two-sided alpha-level of 5%. Assuming a drop-out rate of 10%, 54 patients will be enrolled in this study.

### Secondary endpoints

As secondary endpoints, we consider functional outcomes by means of the Lysholm scale, the Tegner score, and the International Knee Documentation Committee evaluation form (IKDC) in its German translation, 2000 revision [18]. Besides disease-specific items, this questionnaire also contains the Short-Form 36 (SF-36) generic health assessment tool. The noted instruments have proven reliability, validity, and responsiveness for use in clinical research.

Confirmatory testing will apply for the primary endpoint only. All secondary endpoints will be addressed in an exploratory fashion.

### Design

SPOT is a patient and investigator blinded, randomised controlled trial conducted at a single academic institution.

Randomisation is carried out in the operating theatre shortly before transplant fixation, with random codes drawn from sealed envelopes. We use block-randomisation with five subjects a block following a computer-generated random list [19].

### Inclusion criteria

Men and women (providing that they are not pregnant) being at least 18 years old are recruited to this trial. Subjects may engage in this study if they

- faced a **first one-sided total or subtotal rupture of the anterior cruciate ligament**, proven either by arthroscopy or MRI-scanning

- had met an acute knee distorsion event likely to have caused the index injury at least six weeks before scheduled repair

- have been physically examined in the ambulatory of the study hospital before assigning an admission date, and were screened and considered suitable to enter this trial by one of the investigators

Also, patients must be able to give voluntary written informed consent, and to comply with the post operative follow up regime

### Exclusion criteria

We exclude patients

- with related lower limb fractures

- with active infection affecting the limb subject to needed treatment

- who have previously took part in this investigation or who are taking part in another clinical investigation

- with contraindications for MRI-scanning (that is, large indwelling orthopaedic implants made of metals others than titanium, or pacemakers)

### Ethical considerations

This protocol and all accompanying documents were approved by the local Institutional Review Board (IRB).

According to IRB recommendations and requirements, information leaflets explicitly note that "a benefit from participation in this trial cannot be guaranteed."

We will stress the principle of randomisation as "treatment assignment by chance, without the possibility of the investigator, other health care professionals involved in this study, or the patient influencing the choice of treatment." We also tell patients that, as long as they keep agreement in participation, they will not know their assigned treatment until the six-month follow-up visit.

We will notify the IRB of any significant changes to the protocol. Also, we will notify the IRB within ten working days of the discovery of any severe adverse events which occur during this investigation.

Confidentiality of subject data will always be maintained. Subject anonymity will be guaranteed and all documentation about a subject (including radiographs) will be kept in secure location.

This investigation strictly adheres to the relevant articles of the Declaration of Helsinki as adopted by the 18^th ^World Medical Assembly in 1964 and its later revisions, as well as to principles of GCP, developed within the Expert Working Group (Efficacy) of the International Conference on Harmonisation of Technical Requirements for Registration of Pharmaceuticals for Human Use (ICH).

### Surgery and rehabilitation

All devices and instrumentations used in this clinical investigation bear the CE mark. They belong to the regular implants used for ACL repair at the study hospital since 2002. Except different graft anchoring, similar treatments apply to patients in both study groups. All participants undergo internationally accepted surgically procedures by a single surgeon (D.C.) with extensive experience in ACL repair using both the BioCryl^® ^screw and RigidFix^® ^cross pins. Also, postoperative care and rehabilitation programs do not differ from those employed outside a clinical trial.

All repairs are carried out under general anaesthesia, with the patient in a supine position. Perioperative antibiotic prophylaxis comprises 2 g of cefotiam. Patients receive 40 mg of enoxaparin daily for prophylaxis of thromboembolic events until full weight bearing.

The knee joint is accessed through two to three standard portals. Meniscal injuries are addressed with partial resection or repair.

Hamstring tendons are harvested via a small incision over the insertion of the pes anserinus at the anterior medial tibia by a closed tendon stripper, and prepared as triple-stranded grafts.

Tibial and femoral tunnels are drilled to approximate graft thickness (usually 8 to 9 mm) with the use of a guiding wire. Grafts are fixed with the knee in 30° flexion to achieve firm tension.

Postoperatively, the knee is stabilized for three days by a zero-degree splint. Afterwards, flexion is limited to 90° by a Secutec^® ^orthosis for six weeks. Patients are allowed partial weight bearing with walking crutches. Subjects are prescribed intense physical therapy for motion exercise, and to strengthen thigh muscles. Normally, full range of motion and weight bearing is achieved until week 12 after surgery.

Patients in the experimental group have their grafts secured by tibial and femoral RigidFix^® ^pinning. Patient in the control group receive tibial and femoral BioCryl^® ^screws.

### Baseline assessment

Each subject considered eligible for entry into this investigation has the following information and procedures recorded at the pre-investigational examination:

- Demographic details including date of birth and gender

- Medical history, coexisting diseases, and accompanying medication

- Physical examination, including circumferential measurement of both legs at defined landmarks, Lachman and pivot shift tests, KT-1000 arthrometer objectifying of instability, one-legged hop test, Lysholm, Tegner, and IKDC scores, knee and kneeling pain measured by visual analogue scales

- Radiographic examination, including a conventional roentgenogram of the injured knee in anteroposterior and lateral projection, and a preoperative MRI scan according to local standards

### Intraoperative assessments

During surgery, we record procedure details in the electronic CRF. We assess the duration of surgery (from cut to skin closure), and operating theatre time (from induction of anaesthesia to arrival at the recovery room). A clinical knee examination is performed and documented with the subjects under general anaesthesia and relaxation. Arthroscopy findings (accompanying injuries to or degenerative changes of the cruciate or collateral ligaments, menisci, or cartilage) are recorded by video and/ or hard copy images.

We document eventual blood loss, and any other adverse event occurring during surgery.

The responsible surgeon judges the handling of implants and his overall satisfaction with the intraoperative result using five-point Likert scales. We detail any problems or complications on an Adverse Events form.

### Follow-up assessments

Patients are appointed outpatient visits as part of the clinical investigation at 3, 6, and 12 months postoperatively. For study purposes, except quality of life measurements, patients do not undergo any diagnostic or other procedure not belonging to the common repertoire of assessments carried out after ACL repair. Specifically, we avoid invasive procedures, blood sampling, or imaging tests exposing subjects to radiation or contrast agents. The investigators consider the possible burden caused by extra clinical tests negligible.

We assess the following items at the scheduled visits:

#### Three months postoperatively

- Physical examination, including circumferential measurement of both legs at defined landmarks, Lachman and pivot shift tests, KT-1000 arthrometer objectifying of instability, one-legged hop test, Lysholm, Tegner, and IKDC scores, knee and kneeling pain measured by visual analogue scales

- Any complications or complaints raised by the patient, GP/ ambulatory surgeon, or Clinical Investigators

- Resumption of work

- MRI scan according to local standards

#### Six months postoperatively

- Physical examination, including circumferential measurement of both legs at defined landmarks, Lachman and pivot shift tests, KT-1000 arthrometer objectifying of instability, one-legged hop test, Lysholm, Tegner, and IKDC scores, knee and kneeling pain measured by visual analogue scales

- Any complications or complaints raised by the patient, GP/ ambulatory surgeon, or Clinical Investigators

- Resumption of work and leisure activities

- MRI scan according to local standards

Patients and Investigators responsible for follow-up examinations may learn about the assigned treatment after completing the six-month CRF.

#### Twelve months postoperatively

- Physical examination, including circumferential measurement of both legs at defined landmarks, Lachman and pivot shift tests, KT-1000 arthrometer objectifying of instability, one-legged hop test, Lysholm, Tegner, and IKDC scores, knee and kneeling pain measured by visual analogue scales

- Any complications or complaints raised by the patient, GP/ ambulatory surgeon, or Clinical Investigators

- Resumption of work and leisure activities

- MRI scan according to local standards

### MRI studies

Radiological evaluation comprises

- Tunnel widening

- Impingement

- Transplant sufficiency, morphology of transitional areas and tendon-bone-interfaces

- Degree of degradation of screws and pins

- Degree of inflammation (synovitis) and effusion

- Presence or progression of arthrofibrosis

- Changes in cartilage and meniscal morphology

### Physical examination

Before physical examination, both knees are prepared by applying opaque dressings to hide scars and to blind the examining doctors for the managed side.

Examination is performed independently by two of three board-certified surgeons (D.S., K.B., V.T.) who had not operated on any of the patients in the study. The responsible surgeon (D.C.) conducts a third clinical examination after completion of the case report forms. We document his examination findings separately and consider them as the diagnostic reference standard. We assess both interobserver agreement by kappa statistics and the accuracy of measurements taken by independent observers comparing with those of the responsible surgeon.

### KT-1000 arthrometer testing

Objective translation measurements comprise a defined load (89 N). We measure the anterior translation of the injured and healthy side (in mm), as well as the difference between both knees.

### Safety assessment and reporting of adverse event

We define an adverse event as 'any undesirable clinical instance in a subject whether it is considered treatment related or not'. In addition, an adverse device effect, undesirable side effect, is defined as 'a device related adverse event'.

A record of all adverse events, including details of the nature, onset, duration, severity, relationship to the device, relationship to the operative procedure and outcome, will be made on the relevant section of the subject's CRF. The subject will be questioned about any adverse event at each later follow-up assessment visit.

An adverse event or an adverse device effect may be mild, moderate or severe and are usually unexpected.

A severe adverse event or adverse device effect is defined as any experience that

- is fatal or life-threatening

- is permanently disabling

- needs or prolongs in-patient hospitalization because of a potential disability, danger to life or forces an intervention

All severe adverse events or adverse effects which occur during this investigation must be and will be reported immediately by telephone or facsimile to

Bundesinstitut für Arzneimittel und Medizinprodukte, Kurt-Georg-Kiesinger-Allee 3, 53175 Bonn, Phone: ++49 30 228 207 30, Fax: ++49 30 228 207 5207

### Data management

For data collection, we set up a Microsoft XP Professional Access^® ^Database, run on a mobile computer separately from the hospital documentation and the intranet. Data collection and storage comply with orders fixed by the data safety board of the Unfallkrankenhaus Berlin, and follow German laws for data safeguard and protection (*Bundesgesetz über den Schutz personenbezogener Daten *[Datenschutzgesetz 2000 – DSG 2000], 17. August 1999, BGBl. I Nr. 1999/165). We ensure data storage for five years.

For study documentation, we assign patients an identification number. Electronic sources do not contain names or addresses of participants. Linking lists are stored in a study folder with copies of adverse events forms.

Since this study runs at a single centre, we do not appoint an external monitor for data handling and management. We regularly (at least twice a month) check datasets for consistency, completeness and plausibility.

### Statistical analysis

We will conduct all analyses following the intention to treat principle (that is, patients will be evaluated as randomized).

We will express measurements as means, medians or proportions with their proper distribution indices (that is, standard deviations, ranges, and interquartile ranges). In case of skewed distributions, normalizing will be achieved by logarithmic transformation, where necessary.

As pointed out earlier, we will address only the primary hypothesis in a confirmatory fashion, whereas all other results will be evaluated in a plain exploratory intent.

We will employ the student's t-test for independent samples to test for the difference in anterior laxity between both fixation methods at six months of follow-up.

For secondary endpoint analysis, we will calculate cross tables, 95% confidence intervals for normally or binomially distributed data, and differences in means, proportions, and ratios. In case of obvious benefits or harms with either device in a certain subgroup of patients, we will use stratified analyses (for example, according to Mantel-Haenszel).

Where statistically and/ or clinically sound, we will consider linear and logistic regression analyses, or more sophisticated regression models for correlated data (for example, generalized estimating equations). We will, however, respect the small sample size of this study, and limit statistical analyses to the necessary minimum.

In case of missing data, we will use both a last observation carried forward approach, and imputation methods by regression or semi-Bayesian modelling. Separate analyses will be performed for raw and modelled data.

## Discussion

After ACL repair, most patients rarely recognize slightly weakened anterior knee stability in everyday life. However, subjects with a high recreational and sporting activity and physically strenuous professions often suffer from recurrent events of giving-way, especially on hastened movements. This poses a high risk for secondary knee injury. Of note, muscular training cannot compensate for residual laxity, outbalancing the anticipated benefit from surgical repair.

Thus, attempts to optimize the surgical technique may be valuable. Currently, surgeons performing ACL reconstruction use screws, pins, buttons, and cramps for graft fixation because of individual preference, or institutional orders. The latter are chiefly driven by cost considerations. For example, the purchase price of a RigidFix^® ^tray is ten times higher than that of BioCryl^® ^screws. Obviously, the more expensive implant must prove a distinct *clinical *advantage over the common one to justify its further use. Unfortunately, there is lack of robust evidence on the effectiveness of either fixation method beyond laboratory and animal experiments.

Although conceptually impressive, there is no comparative study that proved a clinically measurable advantage of RigidFix^® ^over screws. The investigators consider the equipoise principle fulfilled, since it is unclear whether screw or pins lead to better long-term stability, or show any measurable differences at all. We hope that the results from this pragmatic study can clarify this issue.

## Competing interests

MW, DS and AE have worked as independent scientific consultants for DePuy^® ^International, and received project-related funding that does not apply to this work. No support in any form was provided, or will be provided by third parties to set up the trial protocol, or to conduct this study. This study aims at investigating biomechanical principles, not certain implants.

The members of the SPOT Group have no financial or non-financial competing interests in this work.

## Authors' contributions

DS drafted the manuscript. KB and GM edited the manuscript.

DC is in charge of all surgical procedures. DS is responsible for statistical analyses.

All authors participated in the design of this study, and approved the final manuscript.

## Pre-publication history

The pre-publication history for this paper can be accessed here:


